# Building states’ capacity to address dementia

**DOI:** 10.1093/geront/gnaf226

**Published:** 2025-10-01

**Authors:** John Shean, Kina White, Kristen Felten, Victoria O’Connor, Elma Johnson, Meghan Fadel

**Affiliations:** Healthy Brain Initiative, Alzheimer’s Association, Chicago, Illinois, United States; Office of State Health Planning and Research, Mississippi State Department of Health, Jackson, Mississippi, United States; Office on Aging, Wisconsin Department of Health Services, Madison, Wisconsin, United States; Alzheimer’s Disease and Related Disorders Program, Rhode Island Department of Health, Providence, Rhode Island, United States; School of Public Health, BOLD Public Health Center of Excellence on Dementia Caregiving, University of Minnesota-Twin Cities, Minneapolis, Minnesota, United States; Healthy Brain Initiative, Alzheimer’s Association, Chicago, Illinois, United States

**Keywords:** Alzheimer’s disease, Public health, Capacity-building, Public health intervention

## Abstract

One of the principal objectives of the Building Our Largest Dementia (BOLD) Infrastructure program is to elevate dementia as a public health priority in state, local, territorial, and tribal health departments across the United States. Since 2020, the BOLD Program, through the stewardship of the Centers for Disease Control and Prevention, has supported 45 state and other health departments throughout the United States to refine and implement strategic public health action plans to address dementia that focus on risk reduction, early detection, and caregiving using the framework of the Healthy Brain Initiative Road Map Series. Following an overview and description of the extent of BOLD’s reach, we will highlight several exemplars from individual states’ work in advancing dementia as a public health priority, including efforts to engage local Geriatric Workforce Enhancement Programs to facilitate age-friendly local healthcare systems; working with diverse faith-based communities to disseminate risk reduction strategies; and supporting and training county staff to better meet the needs of dementia caregivers in their respective communities, among others. The resources, opportunities, and challenges to initiate key public health actions to address dementia vary widely across state, local, territorial, and tribal communities, and the current article will demonstrate how Centers for Disease Control and Prevention’s BOLD Program has begun to address this rich diversity throughout the United States.

Over the past 20 years, the public health field has increasingly dedicated resources, time, and staff to address brain health, dementia, and caregiving. The Building Our Largest Dementia (BOLD) Infrastructure for Alzheimer’s Act (Building Our Largest Dementia Infrastructure for Alzheimer’s Act, 2018) aims to strengthen public health infrastructure to better support people living with dementia and the families and communities who care for them. Since 2020, the Centers for Disease Control and Prevention (CDC) has supported 45 health departments and tribal health organizations to refine and implement public health action plans that focus on reducing the risk of cognitive decline, promoting early detection and diagnosis of dementia, and supporting the safety and quality of care for people living with dementia and their caregivers. Informed by the Healthy Brain Initiative (HBI) Road Map Series, the energy and resources this key legislation provides have significantly expanded governmental capacity to address dementia.

## BOLD Program goals and objectives

The Building Our Largest Dementia Infrastructure for Alzheimer’s Act (BOLD Act) directs the CDC to enhance the public health infrastructure to address dementia. As part of this authority, CDC developed the BOLD Program (BOLD Infrastructure for Alzheimer’s Act, 2024)—a multi-year cooperative agreement program to provide funding to state, local, and territorial health departments and tribal health organizations to develop and implement coordinated public health strategies to reduce the risk of cognitive decline, enhance early detection and diagnosis, prevent avoidable hospitalizations, and support family dementia caregivers in their jurisdictions. BOLD Program recipients work along three prevention levels:

1. Primary (e.g., dementia risk reduction)
2. Secondary (e.g., early detection and diagnosis, linkages to treatment, care, and services)
3. Tertiary (e.g., prevention and management of comorbidities leading to preventable hospitalizations and poor health outcomes, and caregiving for persons with dementia).

Broadly, BOLD Programs aim to improve public health capacity to address brain health, dementia, and caregiving; build and sustain public health infrastructure to support these efforts; and work cross-divisionally to enhance outcomes. They accomplish this through various activities, including increasing awareness and understanding of dementia and related topics among the general public and the professional workforce and increasing the number of community–clinical linkages among health care systems, existing community-based services and supports, public health agencies, and other community-based organizations.

BOLD Programs focus attention on social and structural determinants of health to promote health equity and utilize the HBI Road Map Series to guide this work: the HBI *State and Local Road Map for Public Health, 2023–2027* (Alzheimer’s Association and Centers for Disease Control and Prevention, 2023) and the HBI *Road Map for American Indian and Alaska Native Peoples* (Alzheimer’s Association and Centers for Disease Control and Prevention, 2024). These guidebooks outline 24 actions for state, local, and territorial health departments and partners and 13 actions for tribes and tribal health organizations, respectively.

Each BOLD Program is awarded one of two funding awards based on current jurisdictional capacity and infrastructure. Jurisdictions with strong public health infrastructure and established Alzheimer’s disease and related dementias (ADRD) strategic action plans receive Enhanced Capacity (later renamed Component 2) awards that focus solely on implementing actions included in their strategic plan and the HBI Road Map Series. Jurisdictions that need to build and establish jurisdiction-wide public engagement infrastructure receive Core Capacity (later renamed Component 1) awards that first focus on building recipient capacity and developing their ADRD strategic action plans before moving to support implementation.

## BOLD Program reach and implementation

**Figure 1.**
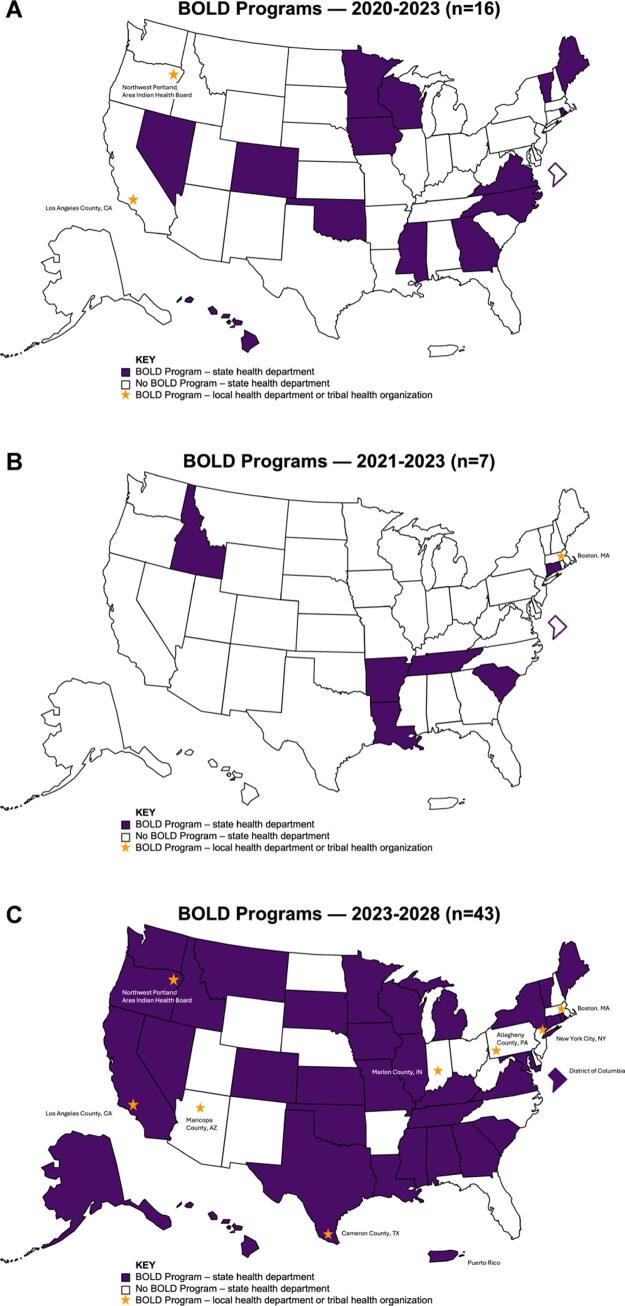
Maps of the 2020–2023, 2021–2023, and 2023–2028 BOLD Programs. (A) BOLD Programs, 2020–2023 (*n* = 16); (B) BOLD Programs, 2021–2023 (*n* = 7); (C) BOLD Programs, 2023–2028 (*n* = 43).

Since 2020, 44 state, local, and territorial health departments and 1 tribal health organization have received a BOLD Program Award (see Figure 1). The BOLD Program has nearly doubled in size and reach with the most recent round of awards. In 2020, the CDC funded the first round of BOLD Programs, awarding 14 state health departments, 1 local health department, and 1 tribal health organization. In the second round, CDC funded six additional state health departments and one additional local health department in 2021. In September 2023, CDC awarded the third round of BOLD Programs, comprised of 33 state health departments, 7 local health departments, 2 territorial health departments, and 1 tribal health organization. In the third round, 15 state health departments, 5 local health departments, and 2 territorial health departments were first-time BOLD Program Award recipients.

All BOLD Programs convene and maintain jurisdiction coalitions composed of community members and leaders. BOLD coalitions include representatives from governmental public health and aging services agencies, subject matter experts, people with lived experience, health care providers, academics, policymakers, and health system personnel, among many others. These coalitions help develop, guide, and implement jurisdictional ADRD strategic action plans. For each program, these strategic action plans may serve multiple purposes—they may be used as state Alzheimer’s plans or integrated or aligned with other strategic plans, such as health improvement plans, multi-sector plans on aging, and agency-specific plans.

Jurisdictional ADRD strategic action plans provide structure and recommendations for implementing the HBI Road Map Series and advancing population health outcomes related to brain health, dementia, and caregiving. Nationwide, BOLD Programs have identified actions to:

- Educate the general public about dementia and risk reduction, early detection, and safety and quality of care.
- Educate health care providers and other professionals about dementia and risk reduction, early detection and safety, and quality of care.
- Increase the availability and use of data on cognitive decline, caregiving, and health disparities experienced by highly impacted populations to improve programs and decision-making.
- Enhance sustainability of brain health, dementia, and caregiving public health work.
- Increase the number of community–clinical linkages among health care systems and existing services, public health agencies, and community-based organizations to improve coordination and dementia-related outcomes.

Each coalition develops and sets the recommendations of their jurisdictional ADRD strategic action plans. Each recommendation included within BOLD jurisdictional ADRD action plans reflects their community’s unique strengths and needs, with many recommendations prioritizing populations highly impacted by dementia. Examples from Mississippi, Wisconsin, and Rhode Island demonstrate the unique ways three BOLD Programs are enhancing state infrastructure to improve health outcomes. These three examples showcase diverse and innovative ways BOLD Programs are implementing the public health approach to Alzheimer’s and dementia.

## Working with diverse faith-based communities in Mississippi

The Mississippi Alzheimer’s Disease and Related Dementias Program (MS BOLD) (Healthy Aging, n.d.) within the Mississippi State Department of Health advances strategies for dementia risk reduction through a comprehensive public health approach. With a focus on risk reduction education and mitigation of modifiable risk factors associated with dementia and related chronic conditions, the MS BOLD Program engages faith-based organizations to reach African American communities.

Collaboration is essential to addressing dementia risk. By working with faith communities, the MS BOLD Program leverages the community strengths of social and spiritual connectedness, particularly within African American churches. For many, faith institutions play a vital role in the health and well-being of these communities, including supporting families impacted by dementia and educating their congregations about health and health issues (Epps et al., 2020).

The MS BOLD Program is the first public health agency to partner with and implement the Alter Program (Epps et al., 2022). Alter is a faith-based community outreach program designed to equip African American communities with the tools needed to support families affected by dementia. Over a 2-year period, the Alter Program provides participating faith-based organizations with education, training, and dementia-specific navigation resources. Kick-off meetings introduce the program to the congregation and community, and a needs assessment is conducted shortly thereafter to identify the specific needs of congregants with dementia and the type of support they may need. Beginning in 2023, the MS BOLD Program set out to recruit African American churches annually. As of September 2024, nine local faith-based organizations have joined Alter. These churches serve as vital resources for African American families living with dementia, helping to educate congregations about its impacts and the importance of community support.

A key component of this initiative is the Dementia Friendly Congregation Workshop, designed to empower faith ministry leaders. This workshop equips faith leaders with the tools and resources necessary to create inclusive environments for individuals living with dementia and their caregivers. Participants learn about effective communication strategies, the significance of patience and understanding, and ways to foster a supportive faith community. Through the workshop, faith leaders gain practical skills that enable them to:

1. Recognize Signs of Dementia: Training helps leaders identify early signs of dementia in congregation members, facilitating timely support.
2. Promote Inclusive Activities: Leaders are encouraged to adapt church activities to be more inclusive, ensuring participation from individuals with cognitive impairment.
3. Support Caregivers: The program emphasizes the importance of supporting caregivers, offering respite, and connecting them to community resources.

The Dementia Friendly Congregation Workshop also encourages congregation leaders and members to adopt healthy lifestyles. Facilitating a physical, emotional, and spiritual wellness program that supports holistic care of body, mind, and spirit is core to creating dementia-friendly faith communities. Respite care is also encouraged for people living with dementia and their care partners to create periods of rest or relief. Healthy habits support brain health, and African American faith communities provide a strong foundation for mental and physical wellness.

### Key outcomes

Since joining the Alter Program network, African American faith leaders in urban and rural communities across Mississippi have been equipped with skills and tools to empower and educate their members.

- Education and Awareness: Four of the five participating churches had congregation members participate during the first implementation year. A total of 17 faith leaders engaged in hands-on training with the Alter Leadership Team and the MS BOLD Program. Representatives of the New Imaging Dementia—Evidence for Amyloid Scanning Brain Imaging Study for Memory Loss shared education on better ways to diagnose and care for people of diverse backgrounds experiencing memory concerns using medical brain imaging.
- Faith and Community Outreach: Purple Sunday is a community event designed to increase awareness of ADRD in Black and Latino faith communities. Faith leaders are encouraged to devote a sermon or spread awareness from the pulpit about Alzheimer’s, while attendees wear purple in unity and prepare a resource table with information about the signs of Alzheimer’s, risk factors for prevention, the importance of early detection, and how to access care and support services. Between 2023 and 2024, eight Purple Sunday events have been held, serving 383 faith and community members. Additionally, participating Alter churches have hosted health and wellness fairs to offer health information, including education about dementia. A total of 215 individuals have been reached through these events.
- Community–Clinical Linkages: Each participating church received a welcome toolkit that includes the *Navigating Alzheimer’s and Related Dementias: A Roadmap for Families* Guide (Alzheimer’s Mississippi et al., 2019). The Mississippi State Alzheimer’s Coalition developed this 200-page resource guide to assist families navigating the disease process. Designed primarily for family caregivers, the guide supplements rather than replaces the recommendations of physicians and other health care professionals providing direct care to individuals living with dementia and their family members. The guide includes a detailed list of clinical and community referral services and connections to a robust caregiver support network.

The MS BOLD Program is expanding its outreach efforts to offer more workshops and recruit additional churches to grow the program’s impact and foster resilience within the community.

By harnessing the power of faith communities, the MS BOLD Program addresses the medical aspects of dementia and nurtures the emotional and social well-being of individuals and families affected by it. Through collaboration and education, the program is paving the way for a more supportive and inclusive environment for all Mississippians affected by dementia.

## Training support staff across Wisconsin

The Wisconsin Dementia Care Specialist (DCS) program (Dementia Care Specialist Program: Replication Resources, 2024) is the state-funded backbone for community-based dementia care services. Administered by the Wisconsin Department of Health Services (WI DHS), the DCS program connects individuals and families with existing public health infrastructure and long-term care services to support them in the communities where they live. The Wisconsin BOLD Program (WI BOLD) has enhanced and expanded access to dementia-specific resources and activities available to communities across the state as part of the DCS program.

DCSs are trained professionals tasked with providing information and support to people living with dementia and their family caregivers and creating and maintaining spaces where people living with dementia can remain active and safe. DCSs may hold full-time or part-time positions and are employed directly by counties and Tribal Nations. In urban areas of the state, DCSs work specifically with African American and Latinx populations. They are available in all counties in Wisconsin (*n* = 72) through the state network of Aging and Disability Resource Centers (ADRCs), and in all federally recognized Tribal Nations in the state (*n* = 11).

The Wisconsin state budget funds the DCS program, and funds are contracted out to individual counties and Tribal Nations by the WI DHS. WI DHS provides ongoing program education, coordination, and technical support. It also convenes and operates a community of practice for all DCSs across the state.

Rather than clinical settings, ADRCs are human services agencies that connect individuals and families to a variety of community-based services and supports, including private pay resources, Older Americans Act programming, and access to publicly funded long-term care benefit programs. ADRCs also provide linkages to aging and disability services, including those related to cognitive concerns.

An essential feature of the DCS program is the availability of home visits rather than a phone call or requiring the customer to travel to the ADRC or Tribal Nation partner agency office. Due to challenges with transportation and winter weather, the ability for the DCS to come out to the home is a crucial feature of the program. Home visits give staff members the opportunity to learn more about the person and their daily challenges. They also help overcome stigma or embarrassment that may prevent an individual from visiting a county or tribal agency or having to navigate complex information in a phone conversation.

### Three pillars of the dementia care specialist program

The DCS program includes three pillars of service that work together to provide a web of support for people living with dementia and their caregivers in the community. Individuals living with dementia or their families may access one or all pillars throughout the course of dementia.

The first pillar provides local public health workforce development, including basic training for all frontline and support staff in recognizing that a customer may have or be caring for someone living with dementia. Training includes the signs of dementia, how to make referrals to locally available supports, and, when necessary, providing case consultations for families. This training is also available for other county-based programs that serve older adults, including adult protective services workers, crisis response and first responder programs, public health offices, and senior centers. WI DHS supports these local efforts through the DCS community of practice. WI DHS provides regular meetings and training sessions to share updates and lessons learned across the DCS program, distribute resource materials, provide connection to the latest research about dementia, and encourage the sharing and growth of new staff through a mentorship program.

WI DHS administers a train-the-trainer program for county-based DCSs to educate ADRC staff on screening for memory concerns. As part of this program, all customer-facing staff within the ADRC receive this training from the DCS. Each DCS oversees the memory screening program to ensure that it is a standard service provided at all ADRCs and that the program encourages conversations about cognitive concerns, connects families to diagnostic services, improves understanding of dementia, enhances awareness of resources, and modifies behavior as a result of education received during the screening.

In the second pillar of the DCS program, DCSs act as catalysts to bring together local coalitions leading dementia-friendly activities. Social isolation and loneliness are concerns not only for people living with dementia but also for their caregivers. Providing opportunities for people living with dementia and their families to continue to participate in social activities and community life is an important feature of a dementia-friendly community.

Memory cafes are dementia-friendly places where people naturally gather for social outings. These may include restaurants, parks, nature centers, senior centers, community centers, religious organizations, VFW Posts, or anywhere people gather and socialize. Memory cafes are not support groups; instead, they are opportunities for people living with dementia and their caregivers to socialize in a public place for a short time free from stigma. DCSs provide support for the development of memory cafes in their jurisdictions, including to organizations such as museums that operate their own programming to assist the public in accessing their spaces.

Dementia-friendly business training provides free customer service training for businesses. Sessions are 20–30 min designed to train businesses in how to support their customers living with dementia and their families. Staff taking the training learn how to recognize cognitive issues among customers, offer support, and maintain them as customers. Banks, e.g., are critical for independent living and receive specialized training, such as what staff can do in cases of suspected dementia and possible financial abuse.

The third pillar of the DCS program is direct engagement with individuals and families. DCSs dedicate much of their time to this third pillar, working directly with customers to better understand their situation, provide education, discuss possible options and resources that meet their specific circumstances, and develop a care plan, including pre-crisis planning. DCSs offer evidence-based and evidence-informed programs for caregivers and people living with dementia.

In 2024, the WI BOLD program partnered with the University of Wisconsin Alzheimer’s Institute to evaluate the DCS program. Several DCSs were invited to help inform the development of the evaluation plan and structure. The evaluation will assess program performance and its unique features, as well as analyze outcomes. The final evaluation analysis is expected at the end of the BOLD Program award in 2028.

## Training primary care sites in Rhode Island

Rhode Island used its BOLD Program Award to establish the Rhode Island Alzheimer’s Disease and Related Disorders Program (RIADRDP; Alzheimer’s Disease and Related Disorders Program, n.d.) within the Rhode Island Department of Health in 2020. Guided by the statewide Alzheimer’s Disease and Related Disorders strategic plan (Rhode Island Advisory Council on Alzheimer’s Disease and Related Disorders, 2024) and with support from the legislatively mandated Advisory Council, the RIADRDP promotes brain health education, informs policymakers, educates health care workers, and utilizes multiple data sources to guide statewide decision-making.

In the spring of 2023, RIADRDP partnered with the Care Transformation Collaborative of Rhode Island and the Rhode Island Geriatric Education Center to launch a quality improvement initiative to enhance the capacity of Rhode Island primary care sites to provide age-friendly care, achieve national recognition through the Institute for Healthcare Improvement (IHI), and address the needs of dementia caregivers.

The IHI Age-Friendly Health System 4M model (Age-Friendly Health Systems, n.d.; Manning et al., 2023) is an evidence-based approach to care for adults 65 and older. The 4Ms stand for: What Matters, Mentation (including cognitive assessment and depression screening), Medication, and Mobility. This care model focuses on wellness, functional ability, priorities, and strengths rather than disease. Primary care and hospital settings can institute this model.

The RIADRDP used the MIPS 288 Quality Improvement Measure: *Education and Support of Caregivers for Patients with Dementia* (American Medical Association, 2023) to prioritize and track support for dementia caregivers. Each participating primary care site is responsible for identifying dementia caregivers, documenting their needs, and integrating an education and referral-to-service process for family caregivers into its clinical workflow.

The initiative included two successive components: an ECHO (Extension for Community Healthcare Outcomes; Arora et al., 2008) learning series and a quality improvement initiative.

### ECHO learning series and quality improvement initiative

In spring 2023, RIADRDP and partners created a six-session ECHO series, *Approaches to Dementia Care: Building Blocks for Becoming Age-Friendly.* ECHO is an educational model that creates virtual learning communities of healthcare professionals and subject matter experts through an all-teach, all-learn approach. A team of subject matter experts—including Rhode Island primary care providers, behavioral and allied health providers, and staff from the Alzheimer’s Association, Rhode Island Chapter—helped develop the series. Participants learned about the 4M model and components of comprehensive dementia care and were provided with information on resources and support for caregivers.

The ECHO series launched between April and June 2023. While most participants were interdisciplinary professionals representing the care continuum, four primary care sites attended and provided insight into the ECHO series. To encourage completion of the entire series, participants who attended five out of the six sessions and were willing to present a case study were eligible for a $250 stipend. Between 24 and 33 attendees participated in each session, and 90% of survey responses indicated that participants were highly satisfied with each session.

To support the transition from learning to action, a quality improvement initiative—*The Next Building Block: Implementing the 4M Age-Friendly Framework for Better Care of Older Adults and People Living with Dementia*—was launched to support primary care sites that completed the ECHO series. Five sites applied and were selected for participation. The 6-month initiative began in the fall of 2023 and provided $6,000 to sites to test workflows for providing the 4Ms of age-friendly care and supporting caregivers of people with dementia.

Each site met monthly with a quality improvement facilitator to review progress, discuss next steps, address barriers, help design and implement quality improvement activities, and facilitate connections with community partners. Facilitators also helped the sites identify areas of need and facilitate clinical–community linkages by connecting them with community resources, such as United Way/the Point (Rhode Island’s ADRC) and the Alzheimer’s Association, Rhode Island Chapter.

The sites attended three virtual collaborative sessions throughout the 6 months. During these sessions, subject matter experts provided information about the 4M model and how to align care with what matters most to the patient. The sessions also allowed participants to learn from each other as they presented their quality improvement projects and discussed their successes, challenges, and innovations moving through the initiative. At the final learning session, each site shared its most meaningful activity, strategies for sustainability, improvements over time, and overall experience in the program.

### Key outcomes

Evaluation surveys of the participants upon conclusion of the 6 months identified several key benefits of the quality improvement initiative.

- Caregiver Identification: All five sites created new workflows to identify dementia caregivers. Over 6 months, the five sites identified 170 dementia caregivers and noted this clearly in the corresponding patient’s electronic medical record. One site reported, “Identifying the caregiver, ensuring all staff have access to this information, and implementing a caregiver education workflow has enhanced our care for patients with dementia.”
- Clinical-Community Linkages: The Alzheimer’s Association was invited to discuss community resources at one of the monthly practice facilitation meetings. As a result, two of the five sites included a direct referral to the Alzheimer’s Association Dementia Care Coordination Program in their workflow, and one site assigned a community health worker to oversee and maintain this referral process. Collectively, the sites made 12 referrals during the 6-month initiative.
- Team-Based Care: All five sites noted improvements in leveraging team-based care during the 6-month initiative. One site stated, “We were able to collaborate more closely and understand each other’s roles by engaging as an interdisciplinary team.”
- Pre/Post Assessment Scores: All five sites completed a baseline and post-initiative assessment to evaluate their adoption of the MIPS measure and the 4Ms model. Compared with baseline, each site showed improvement across the What Matters, Mentation, and Mobility measures (Medication scored high in both pre- and post-assessment) upon completion of the initiative.

As a result of this quality improvement initiative, all five sites received Level One recognition as an Age-Friendly Health System. The initiative’s success has led to the program’s expansion with additional primary care sites joining the learning community. A new ECHO series focused on supporting people living with dementia and their caregivers took place in the spring of 2024, and a new quality improvement cycle began in the fall of 2024. The current cycle will open both to sites that are new to the concept of age-friendly care and those ready to expand their existing age-friendly processes.

## Conclusion

The BOLD Act is establishing and strengthening our national public health infrastructure to improve population health outcomes on brain health, dementia, and caregiving in jurisdictions around the country. By dedicating public health resources, time, and staff, the BOLD Program has mobilized state, local, and territorial health departments and tribal health organizations to address dementia. The above state examples show how public health can develop and implement comprehensive public health strategies to reduce dementia risk, promote early detection, and ensure quality care through community engagement and data-driven decision-making. These successes also underscore the importance of collaboration among health departments, community-based organizations, providers, and people with lived experience of dementia and caregiving. With guidance from the HBI Road Map Series, BOLD Programs nationwide are prioritizing health equity and addressing the diverse needs of individuals and families highly impacted by dementia among diverse communities.

Moving forward, public health agencies at the federal, state, local, territorial, and tribal levels must work to sustain and expand these efforts as the need in communities continues to grow. The nine outcomes of the HBI Road Map Series provide the framework for BOLD Programs and all public health partners to work toward collective impact. The continued success of the BOLD Program will not only benefit people living with dementia, their caregivers, and their families but also strengthen the overall health of communities nationwide.

## Data Availability

The authors do not report data, and therefore, the pre-registration and data availability requirements are not applicable.
